# Differentiation of Human Bone Marrow-Derived Mesenchymal Stem Cells into Insulin-Producing Cells: Evidence for Further Maturation In Vivo

**DOI:** 10.1155/2015/575837

**Published:** 2015-05-12

**Authors:** Mahmoud M. Gabr, Mahmoud M. Zakaria, Ayman F. Refaie, Sherry M. Khater, Sylvia A. Ashamallah, Amani M. Ismail, Sawsan M. El-Halawani, Mohamed A. Ghoneim

**Affiliations:** ^1^Department of Biotechnology, Urology and Nephrology Center, Mansoura 35516, Egypt; ^2^Department of Nephrology, Urology and Nephrology Center, Mansoura 35516, Egypt; ^3^Department of Pathology, Urology and Nephrology Center, Mansoura 35516, Egypt; ^4^Department of Immunology, Urology and Nephrology Center, Mansoura 35516, Egypt; ^5^Department of Urology, Urology and Nephrology Center, Mansoura 35516, Egypt

## Abstract

The aim of this study was to provide evidence for further in vivo maturation of insulin-producing cells (IPCs) derived from human bone marrow-derived mesenchymal stem cells (HBM-MSCs). HBM-MSCs were obtained from three insulin-dependent type 2 diabetic volunteers. Following expansion, cells were differentiated according to a trichostatin-A/GLP protocol. One million cells were transplanted under the renal capsule of 29 diabetic nude mice. Blood glucose, serum human insulin and c-peptide levels, and glucose tolerance curves were determined. Mice were euthanized 1, 2, 4, or 12 weeks after transplantation. IPC-bearing kidneys were immunolabeled, number of IPCs was counted, and expression of relevant genes was determined. At the end of in vitro differentiation, all pancreatic endocrine genes were expressed, albeit at very low values. The percentage of IPCs among transplanted cells was small (≤3%). Diabetic animals became euglycemic 8 ± 3 days after transplantation. Thereafter, the percentage of IPCs reached a mean of ~18% at 4 weeks. Relative gene expression of insulin, glucagon, and somatostatin showed a parallel increase. The ability of the transplanted cells to induce euglycemia was due to their further maturation in the favorable in vivo microenvironment. Elucidation of the exact mechanism(s) involved requires further investigation.

## 1. Introduction

In the year 2000, it was estimated that 150 million people were affected by DM, and this number is expected to double in 2025 [1]. For type 1 diabetes, maintenance of appropriate glycemic control using exogenous insulin is possible but imposes a burden on patients. Transplantation of an intact pancreas as well as isolated pancreatic islets is ideal alternative. However, the shortage of cadaveric organs and the need for immunosuppression are limiting factors [2]. Type 2 diabetes can be treated initially using oral medications, but eventually 27% of patients become insulin-dependent. Of these, less than half achieve the recommended HB A_1C_ level [3]. Recent progress in the field of regenerative therapies provides the potential for the generation of surrogate *β*-cells from stem cells derived from a variety of sources. Human embryonic stem cells (h-ESCs) can be expanded and differentiated into all cell types, including insulin-producing cells (IPCs) [4–6]. However, the use of these cells is hampered by ethical considerations as well as by practical issues, such as the lack of available embryos, difficulties with the generation of immunocompatible cells, and the risk of teratomas formed via proliferation of residual undifferentiated cells. The successful reprogramming of human somatic cells into a pluripotent state (iPS cells) would facilitate the production of patient- and disease-specific stem cells [7]. The successful differentiation of these cells into IPCs has been reported [8] However, the teratogenic potential of iPS cells remains alarming [9]. IPCs can also be obtained from stem cells of the umbilical cord or its blood under defined culture conditions [10, 11]. This source has the advantage of providing a large potential donor pool. Nevertheless, the risks of rejection and the need for immunosuppression are important drawbacks. Mesenchymal stem cells (MSCs) display a high capacity for self-replication, thereby providing a large number of autologous cells while avoiding the limitations of ethical issues, organ availability, and allogenic rejection. MSCs derived from various tissues were utilized in an attempt to differentiate them into IPCs. Bone marrow [12–14] and adipose tissue [15] are among several other tissues that have also been used to generate IPCs. Although the use of MSCs as a source for surrogate *β*-cells is very attractive, the most successful differentiation protocols have produced only a modest number of functional IPCs [16]. In a previous study, we reported that the proportion of IPCs at the end of the in vitro differentiation of human bone marrow-derived MSCs (HBM-MSCs) was less than 5% [14]. In another study, the efficiency of three different differentiation protocols was compared. Again, the yield of functional IPCs was meager and comparable between the three evaluated protocols [17]. Despite these disappointing findings, the produced cells induced euglycemia after transplantation into diabetic nude mice [14, 18, 19]. The aim of the current study is to provide an explanation for this paradoxical observation.

## 2. Materials and Methods

### 2.1. Retrieval of Human Bone Marrow Cells

The required approvals for all the procedures in this study were obtained from the ethical committee of the University of Mansoura. Bone marrow aspirates (BMAs) were collected in heparin from the iliac crests of three consenting donors. All donors were type II, insulin-requiring diabetic patients.

### 2.2. Isolation and Expansion of HBM-MSCs

The BMAs were diluted 1 : 1 in low-glucose Dulbecco's modified Eagle's medium (DMEM, Sigma, St. Louis, MO) layered atop a density gradient (Ficoll-Paque, 1.077 g/mL) (Pharmacia, Uppsala, Sweden) and centrifuged for 20 min at 600 ×g. The cells were collected from the DMEM/Ficoll interface, washed twice in phosphate-buffered saline (PBS), and resuspended in 10 mL of low-glucose complete DMEM (supplemented with 10% fetal bovine serum, (HyClone, Logan, UT), 100 U/mL penicillin, and 100 U/mL streptomycin (Sigma)). One milliliter of BMAs yielded ~1.5 × 10^6^ nucleated cells. The collected cells were cultured in complete DMEM at a density of 5 × 10^5^ cells/mL (10 mL in 25 cm^2^ tissue culture flasks) and incubated at 37°C in a 5% CO_2_ incubator. Aliquots were preserved in liquid nitrogen for subsequent expansion and examination. After 3 days, the nonadherent cells were discarded. The adherent MSCs were cultured to 80% confluence before passaging using trypsin. The cells were resuspended in complete DMEM and reseeded at a ratio of 1 : 2 and cultured for another 8 days to reach 80% confluence. This step was repeated for a second passage. At this point, the cells were spindle-shaped and displayed a fibroblast-like appearance. The samples from each donor were examined in duplicate for the in vitro and in vivo components of this study.

### 2.3. Characterization of the Isolated HBM-MSCs

#### 2.3.1. Phenotyping

HBM-MSCs at passage 3 were trypsinized, centrifuged at 300 ×g for 8 min, and resuspended in PBS at a concentration of 1 × 10^6^ cells/mL. Aliquots of 100 *μ*L were incubated for 30 min in 20 *μ*L of antibodies against CD14, CD45 (FITC) or CD73, CD34 phycoerythrin (PE) or in 5 *μ*L of CD105 PE or CD90 (FITC) (Becton, Dickinson, United States), washed with 1 mL of stain buffer (BD-Pharmingen, United States), and resuspended in 500 *μ*L of stain buffer. The labeled cells were analyzed using an argon ion laser at a wavelength of 488 nm (FACSCalibur, Becton, Dickinson, United States). A total of ten thousand events were obtained and analyzed using CellQuest software (Becton, Dickinson, United States). Control staining using the appropriate isotype-matched monoclonal antibodies was included.

#### 2.3.2. Multilineage Differentiation Potential

HBM-MSCs were induced to differentiate into adipocytes, chondrocytes, and osteocytes using a previously described differentiation protocol [20]. Oil-Red-O was used to stain adipocytes; alcian blue was used to stain chondrocytes; and Alizarin-Red was used to stain osteocytes.

### 2.4. Differentiation of the HBM-MSCs into Endocrine Cells

Differentiation was performed according to a protocol previously reported by Tayaramma et al. [21]. Initially, the cells were cultured for 3 days in serum-free DMEM supplemented with trichostatin-a (TSA) at a concentration of 55 nanomoles (Sigma). Then, the cells were cultured for an additional 7 days in high-glucose (25 millimoles) medium containing a 1 : 1 ratio of DMEM : DMEM/F12 (Sigma). This mixture was supplemented with 10% fetal bovine serum and 10 nanomoles glucagon- (GCG-) like peptide-1 (GLP-1, Sigma).

### 2.5. In Vivo Transplantation Studies in Mice

The ability of the differentiated cells to induce normoglycemia in diabetic nude mice (Swiss Nu/Nu, Charles River Laboratories, Paris, France) was examined following implantation of these cells into the renal subcapsular space. Diabetes was chemically induced using a single dose of 220 mg/kg of streptozotocin (STZ, Sigma). The mice were considered diabetic once the blood glucose levels exceeded 350 mg/dL for 2 consecutive readings. Twenty-nine animals at an average age of 12 weeks were utilized. The diabetic mice were anesthetized via intraperitoneal injection of ketamine (100 mg/kg) and diazepam (5 mg/kg). A total of 1 × 10^6^ of cells obtained at the end of in vitro differentiation were suspended in 20 *μ*L of culture medium and implanted beneath the renal capsule of each mouse. The surviving animals were sacrificed 1, 2, 4, or 12 weeks after transplantation. Before euthanization, blood samples were obtained from the tail vein and measured for blood glucose levels using glucometer strips (Accu-Check, Roche Diagnostics, Basel, Switzerland), serum human insulin, serum human c-peptide, and serum mouse insulin levels by ELISA (DRG Diagnostic, Germany). In addition, an oral glucose tolerance test was performed: 1 g/kg glucose was administered orally via gavage. Blood samples were collected before glucose administration and after 30, 60, 90, and 120 min. Measurements of the glucose and human c-peptide levels were performed on the obtained samples.

The HBM-MSC-bearing kidneys of the euthanized animals were divided into halves. One-half was immunolabeled for histological analysis and to count the insulin-positive cells. The expression of relevant endocrine genes was determined in the other half. The pancreas of these animals was also harvested and immunostained for insulin.

### 2.6. Immunolabeling

#### 2.6.1. Antibodies

An Alexa Fluor 488-conjugated rabbit monoclonal antihuman insulin antibody (Cell Signaling Technology, Denver, United States) was used for flow cytometry. The primary antibodies utilized for immunocytochemistry and immunohistochemistry included mouse monoclonal antihuman insulin, rabbit monoclonal anti-human GCG, rabbit polyclonal anti-human c-peptide (Cell Signaling Technology), and rabbit polyclonal antihuman somatostatin (SST) (Novus Biologicals, Littleton, CO). The employed secondary antibodies were Alexa Fluor 488-conjugated anti-mouse IgG (H + L) and Alexa Fluor 555-conjugated anti-rabbit IgG (H + L) (Cell Signaling Technology).

#### 2.6.2. Flow Cytometry

At the end of in vitro differentiation, the cells were fixed in 4% formaldehyde for 10 min at 37°C, permeabilized using chilled 90% methanol for 30 min and blocked in incubation buffer for 10 min at RT. Then, the cells were incubated in the conjugated antibody for 60 min at RT. Next, the cells were washed with incubation buffer and, after centrifugation, were resuspended in 0.5 mL PBS. The labeled cells were evaluated using a 15 mW argon ion laser at a wavelength of 488 nm (FACSCalibur, Becton, Dickinson, United States). A total of ten thousand events were obtained and analyzed using CellQuest software (Becton, Dickinson). Mouse pancreatic islets served as a positive control.

#### 2.6.3. Immunocytochemistry

Cell preparations were cultured on chamber slides (Nunc, Thermo Scientific, Rochester, NY). Then, the cells were fixed in 4% paraformaldehyde, permeabilized using chilled 100% methanol for 10 min, blocked with 5% normal goat serum for 60 min at RT, and incubated overnight in the primary antibodies at 4°C. Subsequently, the cells were washed with PBS and incubated in the secondary antibodies for 2 hours at RT. Negative controls were performed by omitting treatment with the primary antibody.

#### 2.6.4. Immunohistochemistry

The harvested organs were fixed in formalin and sectioned on coated on positively charged adhesion slides (Citoglas, Citotest Labware manufacturing Co., Haimen, China). Then, the slides were deparaffinized using xylene and a decreasing ethanol gradient. The antigens were unmasked by boiling the slides in 10 millimoles sodium citrate buffer (pH 6.0) and maintaining subboiling temperature for 10 min. The sections were blocked with 5% normal goat serum and incubated overnight in the primary antibody at 4°C. Then, the slides were washed 3 times in PBS and incubated in the secondary antibody for 2 hours at RT. The nuclei were counterstained using DAPI. ImageJ software (developed by NIH) was used to determine the proportion of transplanted cells beneath the renal capsule that intracytoplasmically expressed insulin. To this end, ten fields were randomly selected for cell counting which was carried out by two independent histopathologists. The results from all fields were calculated and expressed as the mean proportion of insulin-positive cells out of the total transplanted cells. In all the above studies, confocal images were captured using a Leica TCS SP8 microscope (Leica Microsystems, Mannheim, Germany).

For immunolabeling of the native pancreas, the primary antibody used was mouse monoclonal anti-insulin (L6B10) (Cell Signaling Technology), and the secondary antibody was the power-stain 1.0 poly-HRP DAB Kit for mouse (Genemed Biotechnologies, California, United States). The sections were examined under light microscopy.

### 2.7. Gene Expression via RT-qPCR

Total RNA was extracted from the undifferentiated cells at the end of in vitro differentiation and from the cells transplanted beneath the renal capsule using the RNeasy plus mini kit (Qiagen GmbH, Hilden, Germany). Three micrograms of total RNA were converted to cDNA using the RT first Strand kit (Qiagen Sciences, Maryland, United States). Custom gene arrays were designed and supplied as the Quantifast Probe Assay (Qiagen Science, Maryland, United States). Gene expression was examined for endocrine hormones (insulin, GCG, and SST), transcription factors (PDX-1, Ngn3, Pax-4, RFX6, and Neurod-1), an endocrine precursor marker (nestin), a glucose transporter (Glut-2), and a pancreatic enzyme (glucokinase (GCK)). Human islets and GAPDH were included as positive and internal controls, respectively. Amplifications were performed in each well using a 25 *μ*L reaction volume consisting of 12.5 *μ*L of 2× TaqMan Master Mix (Quantifast Probe Assay, Qiagen Sciences), 1 *μ*L of cDNA template, 1.5 *μ*L of the primers, and 10 *μ*L of nuclease-free water. The plate was inserted into a real-time thermal cycler (CFX96 Real-Time System, Bio-Rad, United States) that was programmed according to the manufacturer's instructions. The procedure was performed in duplicate for each sample. A mathematical model introduced by Pfaffl was used for the relative quantification of target genes [22]. In this study, gene expression was expressed relative to that in human islets.

### 2.8. Statistical Analysis

Nonparametric data were evaluated using Friedman's test. Post hoc analysis was performed using the Wilcoxon signed-rank test, and the *P* values were corrected using Bonferroni adjustments. A *P* value of <0.05 was considered significant. The mean values were used as a measure of variation. The median values were utilized only if there were extreme observations.

## 3. Results

### 3.1. Characterization of the Cultured HBM-MSCs

At the end of expansion, the cultured cells became spindle-shaped, fibroblast-like cells that arranged in monolayers. Flow cytometry revealed that these cells expressed high levels of CD73, CD90, and CD105 but negligible levels of CD14, CD34, and CD45 (Supplementary Table 1 in Supplementary Material, available online at http://dx.doi.org/10.1155/2015/575837). These cells could be differentiated to form adipocytes, chondrocytes, and osteocytes when the appropriate growth factors were added (Supplementary Figure 1). Accordingly, evidence for their multilineage potential was confirmed.

### 3.2. Functional Evaluation of Differentiated HBM-MSCs

**Figure 1:**
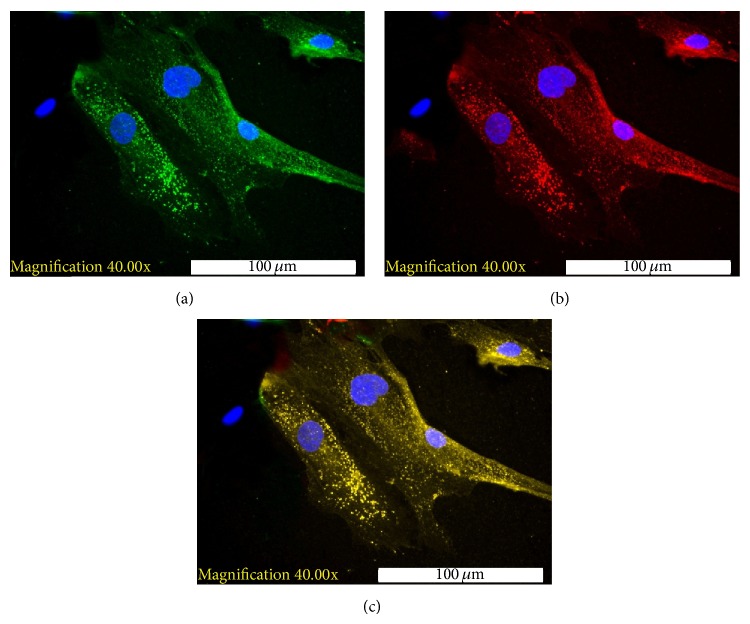
Immunofluorescence staining of differentiated HBM-MSCs ((a) selected field). (a) Positive staining for intracytoplasmic insulin granules (green) with counterstaining for DAPI (blue). (b) Positive staining for c-peptide (red) with counterstaining for DAPI (blue). (c) Electronic merging of the insulin and c-peptide staining. The coexpression of insulin and c-peptide (yellow) was detected in the same cells.

At the end of differentiation, flow cytometric analysis indicated that the percentage of generated IPCs was meager, ranging between 0.12% and 3.4%. The presence of insulin granules within the cytoplasm of the IPCs was detected via immunocytochemistry. Immunostaining for c-peptide was also positive in the IPCs. Coexpression of insulin and c-peptide within the same cells was detected via electronic merging (Figure 1). GCG staining was detected in some of the examined samples. However, positive staining for SST was not detected.

### 3.3. Outcomes of the In Vivo Transplantation Experiments

**Figure 2:**
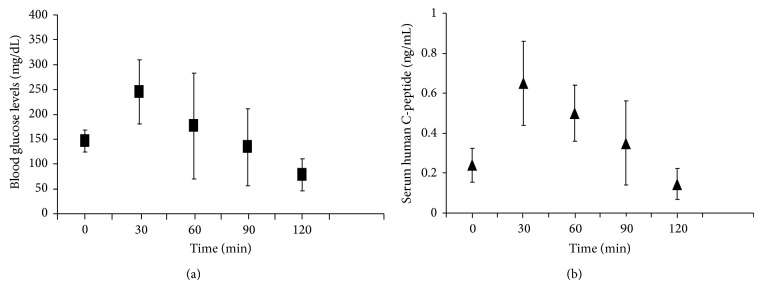
The oral glucose tolerance test performed 4 weeks after transplantation. (a) The blood glucose levels 2, 4, and 12 weeks after transplantation displayed normal patterns. (b) The corresponding serum human c-peptide values demonstrated a similar pattern. This result indicates that the transplanted cells are glucose-responsive and insulin-secreting.

Out of the 29 transplanted animals, 5 mice did not tolerate the surgical procedure. The blood glucose levels of the surviving animals became normalized within a few days after transplantation (4 ± 1.6 days). Thereafter, the animals remained euglycemic throughout the observation period. The serum levels of human insulin and human c-peptide were measurable one week after transplantation, and these values also remained unchanged throughout the observation period. Serum levels of mouse insulin became negligible after induction of diabetes (Table 1). The results of the oral glucose tolerance test were normal. The corresponding c-peptide level measurements indicated parallel changes, providing evidence that the transplanted cells were glucose-responsive and insulin-secreting (Figure 2, Supplementary Table 2).

**Figure 3:**
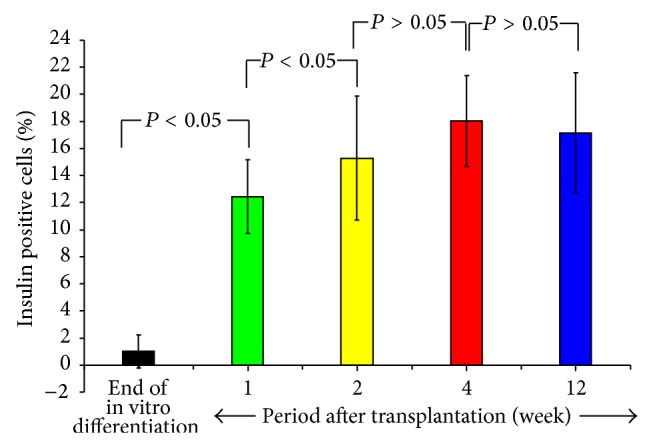
The percentage of insulin-positive cells before transplantation and 1, 2, 4, and 12 weeks after transplantation. Compared to the pretransplantation values, the mean percentage of IPCs (insulin-producing cells) was significantly increased by one week after transplantation. The percentage of IPCs increased progressively, peaking at 4 weeks (18.04 ± 3.3%). There was no substantial change in the percentage of IPCs thereafter.

**Figure 4:**
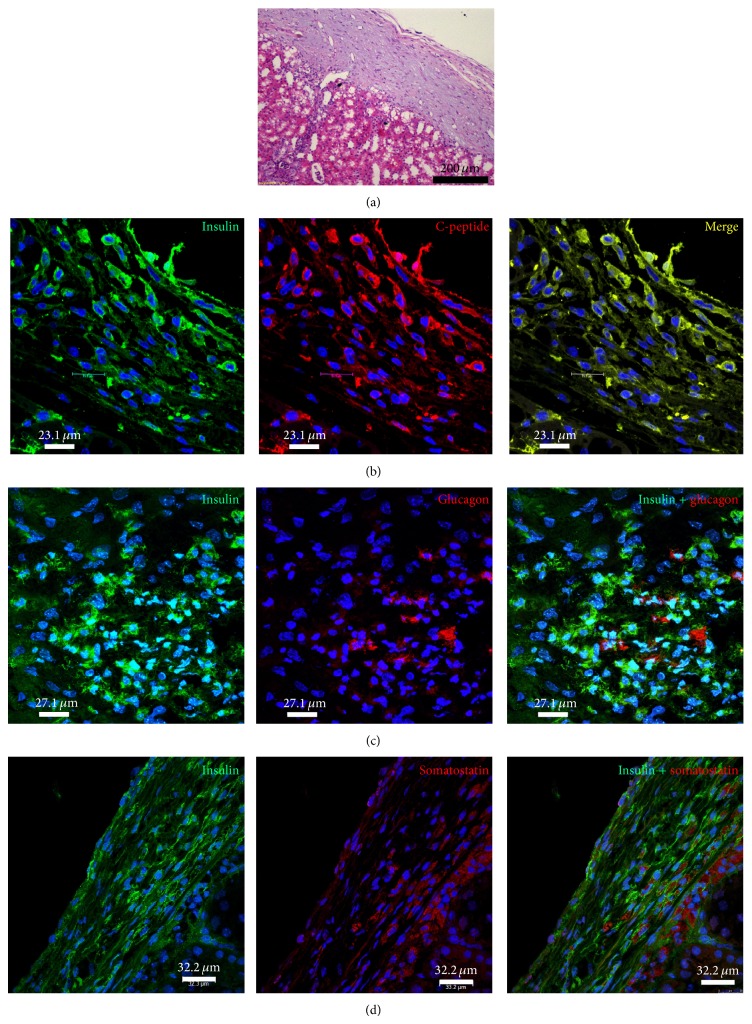
Histology of IPC-bearing kidneys harvested from mice 4 weeks after transplantation. (a) Hematoxylin and eosin staining revealed the implanted cells beneath the renal capsule. (b) Positive immunofluorescence staining for insulin (green) and c-peptide (red). Electronic merging (yellow) reveals the coexpression of insulin and c-peptide in some cells. (c) Positive immunofluorescence staining for insulin (green) and GCG (red). Electronic merging reveals that these two hormones are localized to distinct cell populations. (d) Positive immunofluorescence staining for insulin (green) and SST (red). Electronic merging reveals that these two hormones are localized to distinct cell populations.

**Figure 5:**
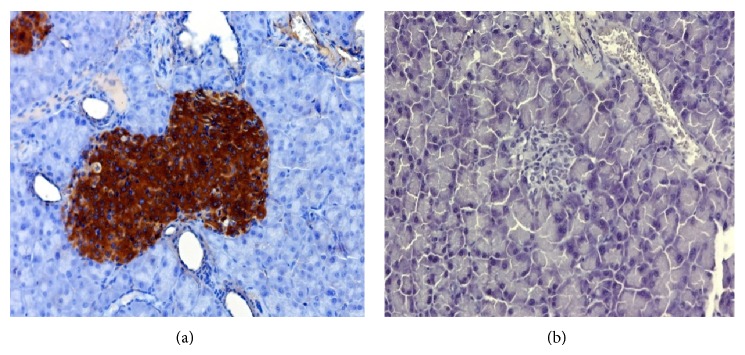
Immunolabeling of the native pancreas (×200). (a) Positive control displaying intense positive staining for insulin within the islets. (b) The pancreas from an STZ-treated mouse 12 weeks after the transplantation of differentiated cells beneath the renal capsule: negative staining for insulin.

Immunohistochemistry of the HBM-MSC-bearing kidneys revealed that the percentage of IPCs increased gradually, peaking at 4 weeks after transplantation (~18%) without any substantial change thereafter (Figure 3, Supplementary Table 3). Again, the coexpression of insulin and c-peptide within the cytoplasm of these cells was confirmed. Positive staining for GCG and SST was detected in some cells that did not display insulin expression (Figure 4). Immunolabeling of the native pancreas of the treated mice was negative for IPCs (Figure 5).

**Figure 6:**
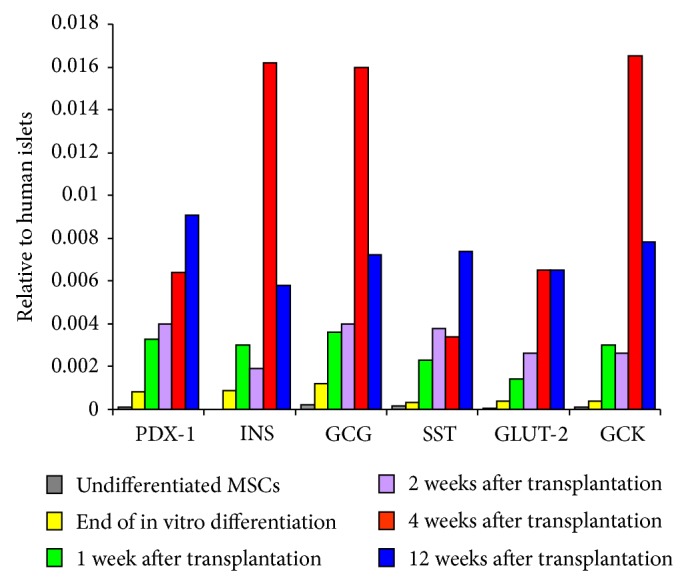
Median relative gene expression levels of the undifferentiated cells at the end of in vivo differentiation and at 1, 2, 4, and 12 weeks after transplantation. At the end of in vitro differentiation, relevant pancreatic endocrine genes were expressed, although at approximately 1/1000 that in human islets. These levels increased by more than 10-fold 4 weeks after transplantation. Insulin, GCG, and GCK gene expression displayed a small but significant decrease at the 12th week. The peak maturation of IPCs occurred at the fourth week. Thereafter, gene expression varied depending on the blood glucose levels at the time of sampling.

At the end of the in vitro differentiation period, relevant endocrine genes were expressed, although at approximately 1/1000 that in human islets. After transplantation, there was a significant increase in the relative gene expression values of the transcription factor PDX1, the endocrine hormones insulin, GCG, and SST, the glucose transporter GLUT-2, and the pancreatic enzyme GCK. This increase peaked by the fourth week after transplantation. At 12 weeks after transplantation, there was a decline in the gene expression of insulin, GCG, and GCK (Figure 6, Supplementary Table 4).

## 4. Discussion

The term MSCs has been popularized by Caplan [23] to refer to plastic-adherent cell preparations isolated from a variety of tissues. Of these, bone marrow and adipose tissue offer distinct advantages in terms of availability, abundance, and the extent of their documentation in the literature. Recently, leading investigators of mesenchymal cell therapy concluded that convincing data to support “the stemness” of these unfractionated plastic-adherent cells are lacking [24]. Therefore, the use of the term mesenchymal stromal cells has been suggested, thus allowing the abbreviation MSCs to be maintained. Several independent studies have demonstrated that MSCs can differentiate into not only mesodermal but also ectodermal and endodermal lineages [25]. Based on these findings, the term multipotent mesenchymal stromal cells appears to be the most scientifically accurate descriptor of this plastic-adherent population. The term “mesenchymal” is maintained to imply the origin, but not the differentiation potential, of these cells [26].

We have shown that the HBM-MSCs utilized in this study met the minimal criteria proposed by the International Society for Cellular Therapy [27]. The feasibility of differentiating HBM-MSCs into IPCs in vitro under defined culture conditions has been reported by many investigators [12, 14, 19, 28, 29]. Based on the results of a previous comparative study, we chose to perform a TSA-based protocol due to its simplicity and the short duration required for differentiation [17].

The blood glucose levels were normalized one week after cell transplantation. The serum levels of human insulin and c-peptide became detectable one week after transplantation, and these values were maintained throughout the observation period. The similarity between the profiles of the glucose levels and the c-peptide tolerance curves provides evidence that these are glucose-responsive and insulin-secreting cells. The percentage of IPCs among the cells transplanted beneath the renal capsule increased over time, peaking at ~18% after 4 weeks. All the relevant endocrine genes, particularly insulin, GCG, and SST, were expressed. Their relative values were significantly higher than those at the end of in vitro differentiation, peaking at 4 weeks after transplantation. The pancreatic enzyme GCK, which is responsible for glucose sensing, and the glucose transporter Glut-2, which initiates glucose-stimulated insulin secretion, were also expressed. A possible explanation for the limited decrease in the gene expression levels of insulin, GCG, and GCK at 12 weeks is that there was no further differentiation after the 4th week of transplantation. Afterward, gene expression may vary depending on the blood glucose levels at the time of sampling. It must be noted that primers utilized for our gene expression studies were designed specifically for detection of human gene sequences. Accordingly, the gene expressed in this study can only be derived from transplanted human cells and not from the mice renal tissues.

We and others have found that the proportion of IPCs at the end of in vitro differentiation is small, irrespective of the employed protocol [13, 14, 17, 19, 30]. Despite this modest yield, we found that these cells induced euglycemia after their transplantation into diabetic nude mice [14, 18, 19]. Some studies suggested that these beneficial effects are due to MSC-mediated *β*-cell regeneration in the pancreas [31]. Such a possibility was firmly excluded by our experiments because histopathologic examination of the harvested pancreas did not reveal any signs of regeneration. Moreover, serum levels of mouse insulin became negligible after induction of diabetes without any change throughout the observation period. It is reasonable to assume that the in vivo milieu contains factors that promote the maturation of the transplanted cells. Several investigators suggested that the source of these factors could be the regenerating pancreas after it had sustained an injury, either toxic or traumatic. Hardikar and Bhonde showed that cytosolic extracts from the regenerating pancreas display the potential to initiate islet neogenesis in STZ-induced diabetic animals [32]. Choi and colleagues utilized an extract from a regenerating pancreas 2 days after a 60% pancreatectomy for the differentiation of rat mesenchymal cells into IPCs [33]. Similarly, Phandis and colleagues reported that paracrine factors secreted from the regenerating pancreas assist in the efficient differentiation and maturation of HBM-MSCs [19]. Further studies to identify the factors secreted during pancreatic regeneration could provide an important tool for achieving the efficient differentiation of HBM-MSCs.

To our knowledge, we have provided the first evidence that the ability of these transplanted cells to cure the diabetic animals was due to an increase in the number of functional IPCs. Directed differentiation in vitro served as an initial step that induced expression of relevant endocrine genes. Subsequently, further maturation of these cells occurred after transplantation under the influence of favorable microenvironmental conditions. The maximal yield of functional IPCs was ~18% at 4 weeks after transplantation, with no further increase thereafter. This result suggests that only a subset of MSCs are capable of trans-differentiation into the pancreatic endocrine lineage.

The identification, sorting, expansion, and subsequent differentiation of this cellular component would result in the production of sufficient IPCs displaying adequate functional capacity. The group of Catherine Verfaillie described a culture system for MSCs that favors the selection of a subpopulation of primitive cells referred to as multipotent adult progenitor cells (MAPCs) [34]. It was shown that these cells can be differentiated into mesoderm, visceral mesoderm (endothelial cells), neuroectoderm, and endoderm [35]. However, because other laboratories were unable to produce MAPCs, their existence was questioned [36]. The intermediate filament protein “nestin” has been detected in several cellular phenotypes during embryonic and adult life. The expression of nestin may reflect the multipotential and regenerative abilities of cells [37]. Kabos et al. described a method for isolating nestin-positive cells from adult bone marrow [38]. Using this method, the successful differentiation of the nestin-positive subset of bone marrow-derived pancreatic endocrine cells was reported by Milanesi and colleagues [39]. However, the superiority of this method over the use of unfractionated cells in terms of the number and/or functionality of the generated IPCs was not provided. Recently, Kuroda and colleagues isolated what they defined as multilineage differentiating stress-enduring (Muse) cells cultured from skin fibroblasts or bone marrow stromal cells [40]. These cells were positive for both CD105, a mesenchymal cell marker, and SSEA-3, a human pluripotency marker. Muse cells are indistinguishable from other MSCs in adherent culture, but when they are transferred to suspension culture, they form characteristic cell clusters that are capable of self-renewal as well as differentiation into all three germ layers. To our knowledge, the differentiation of these cells into IPCs has yet to be reported.

## 5. Conclusions

IPCs can be generated from HBM-MSCs via directed differentiation, although the yield is meager. However, transplanting these cells results in the normalization of blood glucose levels in diabetic animals. Evidence was provided for the further maturation of these cells in vivo. The proportion of IPCs increased by 10-fold 4 weeks after transplantation. All relevant pancreatic endocrine genes were expressed, and their expression levels were significantly increased in vivo. The glucose-tolerance curves and the simultaneously measured c-peptide levels demonstrated that these cells are glucose-responsive and insulin-secreting. Our experiments indicated that only a subpopulation of MSCs are capable of differentiation into the pancreatic lineage. The identification, sorting, and expansion of this cellular subpopulation before differentiation would optimize the yield. However, several questions remain: how long will these cells will retain their function? What is the optimal site for their transplantation? What are the risks of teratogenicity? Once these questions are adequately addressed, meaningful clinical applications can be developed.

## Supplementary Material

Supplementary Material: In figure (1), the multilineage potentials of HBM-MSCs intoadipocytes, chondrocytes and osteocytes had been provided. The cytoflowmetric characterization of HBM-MSCs is shown in table (1). Table 2 shows the glucose tolerance data of the transplanted animals at different time points. In addition to glucose levels, c-peptide values were also determined. The percentage of insulin-positive cells at end of differentiation and following transplantation are given in table 3. Table 4, demonstrates data of the relative gene expression of relevant pancreatic endocrine genes at end of differentiation as well as following transplantation.

## Figures and Tables

**Table 1:** Mean blood glucose and serum human insulin, c-peptide, and serum mouse insulin levels in mice transplanted with HBM-MSCs (human bone marrow-derived mesenchymal stem cells).

	Basal	After induction of diabetes	After transplantation			
			1 week	2 weeks	4 weeks	12 weeks

Blood glucose (mg/dL)	117.5 ± 14.15	359.5 ± 49.6	121.0 ± 24.2	167.2 ± 32.6	146.67 ± 24.1	103.2 ± 17.6
Serum human insulin (*μ*IU/mL)	—	—	29.2 ± 7.57	30.5 ± 6.4	29.64 ± 2.9	32.8 ± 2.86
Serum human c-peptide (ng/mL)	—	—	0.4 ± 0.14	0.33 ± 0.12	0.18 ± 0.39	0.4 ± 0.09
Serum mouse insulin (*μ*IU/mL)	3.65 ± 1.8	—	—	0.4 ± 0.089	0.43 ± 0.11	0.34 ± 0.10
