# Supplementary material for: Differentiation of Human Bone Marrow-Derived Mesenchymal Stem Cells into Insulin-Producing Cells: Evidence for Further Maturation In Vivo
Source: Biomed Res Int. 2015 May 12;2015:575837. doi: 10.1155/2015/575837 (PMC4443784; doi:10.1155/2015/575837)
Supplement: Supplementary file 1 — Supplementary Material: In figure (1), the multilineage potentials of HBM-MSCs intoadipocytes, chondrocytes and osteocytes had been provided. The cytoflowmetric characterization of HBM-MSCs is shown in table (1). Table 2 shows the glucose tolerance data of the transplanted animals at different time points. In addition to glucose levels, c-peptide values were also determined. The percentage of insulin-positive cells at end of differentiation and following transplantation are given in table 3. Table 4, demonstrates data of the relative gene expression of relevant pancreatic endocrine genes at end of differentiation as well as following transplantation. [file 575837.f1.pdf]

Supplementary figure 1.

Multilineage differentiation of HBM-MSCs.

(A) Adipogenesis was detected using oil-Red-O staining.

(B) Chondrogenesis was detected using alcian blue staining.

(C) Osteogenesis was detected using alizarin-red staining

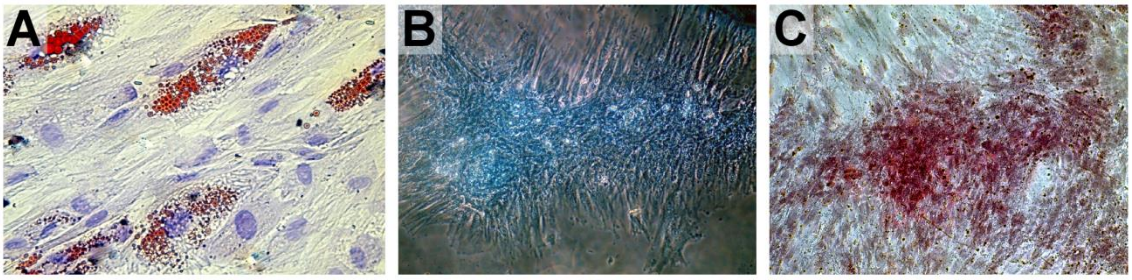

Supplementary Table 1. Flow cytometric quantitation of surface markers of the undifferentiated MSCs

**Donor**	**CD 14**	**CD 34**	**CD 45**	**CD 73**	**CD 90**	**CD 105**
1	1.7	1.2	0.05	99.95	98.05	99.9
2	2.6	0.2	0.1	99.5	99.4	96.4
3	1.9	1.4	0.1	92	99.6	94
4	1.3	2.2	0.3	92	98.4	93
5	2.2	1.5	0.2	91.2	99.5	97.4
6	1.9	2.2	0.1	99.9	99.5	97.7
**Mean±SD**	**1.9±0.5**	**1.3±0.7**	**0.2±0.1**	**96±4**	**99±0.7**	**96±2.5**

Supplementary Table 2: Oral glucose tolerance test (1gm/Kg) of transplanted nude mice (2, 4, and 12 weeks after transplantation)

	**Zero min**		**30 min**		**60 min**		**90 min**		**120 min**	
	Blood Glucose mg/dl	C-peptide ng/ml	Blood Glucose mg/dl	C-peptide ng/ml	Blood Glucose mg/dl	C-peptide ng/ml	Blood Glucose mg/dl	C-peptide ng/ml	Blood Glucose mg/dl	C-peptide ng/ml
2 weeks	196	0.3	277	0.7	189	0.4	63	0.2	77	0.3
	133	0.2	385	0.9	325	0.7	260	0.4	190	0.3
**Mean±SD**	**164.5±44.55**	**0.25±0.071**	**331±76.37**	**0.8±0.14**	**257±96.17**	**0.55±0.21**	**161.5±139.3**	**0.3±0.14**	**133.5±79.9**	**0.3±0**
4 weeks	162	0.18	200	0.5	101	0.4	79	0.2	55	0.09
	131	0.3	291	0.8	252	0.6	189	0.5	101	0.2
**Mean±SD**	**146.5±21.9**	**0.24±0.085**	**245.5±64.3**	**0.65±0.21**	**176.5±106.8**	**0.5±0.14**	**134±77.8**	**0.35±0.21**	**78±32.5**	**0.145±0.078**
12 weeks	83	0.3	167	0.4	121	0.3	45	0.1	45	0.1
	106	0.4	176	0.4	180	0.5	131	0.4	86	0.2
**Mean±SD**	**94.5±16.26**	**0.35±0.07**	**171.5±6.36**	**0.4±0.0**	**150.5±41.71**	**0.4±0.141**	**88±60.8**	**0.25±0.21**	**65.5±29.0**	**0.15±0.07**

Supplementary Table 3: Percentage of in vitro and in vivo insulin-positive cells

	In vitro	1 week after transplantation	2 weeks after transplantation	4 weeks after transplantation	12 weeks after transplantation
Percentage of insulin positive cells	2	8.9	16.4	15.2	14.1
	3.4	14.6	15.7	15.7	18.6
	2.2	14.3	22.8	19.8	16.6
	1.47	12.9	17	16.6	16.5
	2.6	10	20.4	24	17.5
	3	13	13.2	16.96	20
**Mean±SD**	**2.44±0.70**	**12.28±2.32**	**17.58±3.45**	**18.04±3.33**	**17.22±2.02**
**P-Value**	**<0.001**	**0.026**	**0.694**	**0.605**	

Supplementary Table 4: Relative gene expression (Human islets as a control)

	INS	GCG	PDX1	SST	NGN3	PAX4	GCK	RFX6	GLUT2	Neurod1	NES	MafA	MafB	OCT4
**Undiff. MSCs**	0.00000	0.00003	0.00001	0.00010	0.01866	0.00876	0.00018	0.04859	0.00362	0.05892	0.00212	0.00128	0.00682	0.00045
	0.00000	0.00020	0.00014	0.00010	0.00007	0.00012	0.00030	0.00022	0.00028	0.00057	0.00017	0.00082	0.00088	0.00012
	0.00000	0.00024	0.00012	0.00026	0.00020	0.00003	0.00009	0.00017	0.00006	0.00098	0.00138	0.00020	0.09251	0.00007
	0.00000	0.00024	0.00012	0.00026	0.00020	0.00003	0.00009	0.00017	0.00006	0.00098	0.00138	0.00020	0.09251	0.00007
	0.00000	0.00024	0.00013	0.00026	0.00020	0.00003	0.00009	0.00017	0.00006	0.00098	0.00138	0.00020	0.09251	0.00007
	0.00000	0.00006	0.00004	0.00007	0.00007	0.00007	0.00001	0.00050	0.00001	0.00012	0.10162	0.00010	0.01949	0.00007
**Median**	0	0.00022	0.00012	0.00018	0.0002	0.00005	0.0009	0.00019	0.00006	0.00098	0.00138	0.0002	0.056	0.00007
**End of In vitro Diff.**	0.00145	0.00035	0.00043	0.00036	0.00138	0.00625	0.00019	0.00142	0.00018	0.00085	0.00229	0.00035	0.01095	0.00031
	0.00119	0.00129	0.00059	0.00027	0.00035	0.00008	0.00136	0.00140	0.00067	0.00848	0.00214	0.00030	0.01086	0.00014
	0.00142	0.00207	0.00134	0.00235	0.00202	0.00038	0.00026	0.00173	0.00108	0.00853	0.00452	0.00202	0.02456	0.00079
	0.00018	0.00041	0.00062	0.00043	0.00047	0.00015	0.00014	0.00073	0.00037	0.00201	0.00699	0.00060	0.04656	0.00022
	0.00073	0.00114	0.00072	0.00033	0.00145	0.00008	0.00052	0.00263	0.00041	0.00528	0.00134	0.00080	0.01564	0.00015
	0.00003	0.00010	0.00077	0.00005	0.00048	0.00014	0.00096	0.00141	0.00008	0.00125	0.00021	0.00176	0.05711	0.00049
**Median**	0.00096	0.000775	0.00067	0.000345	0.00093	0.000145	0.00039	0.00141	0.00039	0.00364	0.00221	0.0007	0.0201	0.00026
**1 Week After Transpl.**	0.01753	0.02166	0.10124	0.06044	0.07383	0.06595	0.00823	0.07506	0.02126	0.25753	0.13076	0.16053	0.03270	0.03120
	0.00983	0.01006	0.00405	0.00233	0.00270	0.00072	0.01208	0.01246	0.00461	0.01536	0.00342	0.00228	0.00813	0.00030
	0.00337	0.00358	0.00247	0.00159	0.00190	0.00029	0.00354	0.00293	0.00168	0.00821	0.00129	0.00080	0.00646	0.00045
	0.00272	0.00348	0.00251	0.00192	0.00235	0.00029	0.00252	0.00253	0.00120	0.02095	0.00223	0.00450	0.00263	0.00104
	0.00174	0.00130	0.00092	0.00302	0.00335	0.00095	0.00127	0.00680	0.00097	0.01460	0.00424	0.00156	0.00225	0.00019
	0.00150	0.00104	0.00532	0.00231	0.00324	0.00000	0.00125	0.00264	0.00120	0.01632	0.01064	0.00269	0.16885	0.00000
**Median**	0.0030	0.0035	0.0032	0.00232	0.00297	0.0005	0.003	0.00486	0.00144	0.01584	0.00383	0.00248	0.00729	0.00037

	INS	GCG	PDX1	SST	NGN3	PAX4	GCK	RFX6	GLUT2	Neurod1	NES	MafA	MafB	OCT4
**2 Weeks After Transpl.**	0.00370	0.00750	0.01450	0.00740	0.04060	0.00890	0.00440	0.04710	0.01550	0.08600	0.02050	0.01900	0.66910	0.00390
	0.02769	0.02221	0.00597	0.00120	0.00147	0.00057	0.02683	0.03002	0.00892	0.01859	0.00111	0.00147	0.00155	0.00049
	0.00209	0.00180	0.00083	0.00317	0.00204	0.00158	0.00101	0.00180	0.00107	0.01195	0.00193	0.00156	0.03765	0.00025
	0.00068	0.00228	0.00349	0.00299	0.00619	0.00346	0.00267	0.00562	0.00289	0.01366	0.05188	0.03236	0.26840	0.00631
	0.00088	0.00285	0.00227	0.00440	0.00337	0.00252	0.00256	0.00611	0.00235	0.01580	0.02521	0.00335	0.13775	0.00179
	0.00182	0.00511	0.00459	0.00774	0.00410	0.00000	0.00000	0.00411	0.00185	0.07006	0.00440	0.00231	0.08500	0.00193
**Median**	0.0019	0.00398	0.004	0.0037	0.0037	0.002	0.0026	0.0058	0.0026	0.0171	0.012	0.0028	0.111	0.0018
**4 Weeks After Transpl.**	0.00457	0.01247	0.00462	0.04897	0.04234	0.00476	0.01019	0.01439	0.01226	0.00830	0.01109	0.01061	0.00638	0.00208
	0.00773	0.01668	0.00656	0.03263	0.01506	0.00384	0.00295	0.01082	0.00356	0.07379	0.00396	0.01183	0.00776	0.00293
	0.01515	0.01450	0.00571	0.00191	0.00403	0.00101	0.01613	0.01783	0.00511	0.02178	0.00198	0.00470	0.00533	0.00156
	0.01928	0.01977	0.00616	0.00292	0.00568	0.00126	0.02015	0.02631	0.00697	0.03151	0.00263	0.00362	0.00278	0.00160
	0.01729	0.01545	0.00790	0.00371	0.00724	0.00178	0.01908	0.02655	0.00670	0.02968	0.00501	0.01140	0.00719	0.00297
	0.01987	0.01907	0.00739	0.00303	0.00338	0.00078	0.01688	0.01738	0.00642	0.02172	0.00393	0.00397	0.01008	0.00098
**Median**	0.016	0.016	0.006	0.0033	0.0064	0.0015	0.0165	0.0176	0.0065	0.0257	0.0039	0.0076	0.0067	0.0018
**12 Weeks After Transpl.**	0.0041	0.00222	0.00126	0.00148	0.00284	0.00362	0.00118	0.00881	0.00109	0.01188	0.00059	0.00513	0.25618	0.00048
	0.0090	0.00192	0.00347	0.00460	0.00431	0.00056	0.00186	0.02035	0.00226	0.00991	0.00365	0.04579	0.75626	0.00513
	0.0066	0.02418	0.02521	0.01884	0.02628	0.00556	0.01201	0.02352	0.01209	0.11908	0.01795	0.03467	0.01399	0.01136
	0.0048	0.00798	0.01278	0.01342	0.01924	0.00430	0.01278	0.00739	0.00549	0.12500	0.01379	0.01820	0.08077	0.00455
	0.0058	0.00635	0.00534	0.01024	0.01060	0.00609	0.01168	0.00534	0.00744	0.03103	0.00626	0.01038	0.03467	0.00288
	0.0059	0.03013	0.01959	0.00395	0.00000	0.00000	0.00394	0.00491	0.07214	0.01974	0.00377	0.00830	0.05253	0.00000
**Median**	0.00585	0.0071	0.009	0.007	0.007	0.0039	0.0078	0.0081	0.0064	0.0253	0.005	0.014	0.066	0.0037
